# SCHIZOPHRENIA EPIGENETIC AGING PATTERNS REFLECT ALTERED MORTALITY AND CANCER RISKS

**DOI:** 10.1093/geroni/igz038.3266

**Published:** 2019-11-08

**Authors:** Albert T Higgins-Chen, Christiaan Vinkers, Marco P Boks, Morgan E Levine

**Affiliations:** 1 Department of Psychiatry, Yale School of Medicine, New Haven, Connecticut, United States; 2 Amsterdam UMC, Amsterdam, Netherlands; 3 University Medical Center Utrecht Brain Center, Utrecht, Utrecht, Netherlands; 4 Yale University School of Medicine, New Haven, Connecticut, United States

## Abstract

Schizophrenia (SZ) is associated with large increases in all-cause mortality, high smoking rates, and elevated levels of age-associated proteins—suggesting individuals with SZ may experience accelerated rates of biological aging. Yet surprisingly, multiple previous studies found no association between SZ and biological age using Horvath’s epigenetic clock, a well-recognized and validated biomarker of aging based on DNA methylation (DNAm) levels. However, numerous epigenetic clocks have been developed to date, many of which are better indicators of differential lifespan and healthspan than the original Horvath clock. Thus, we hypothesize that these epigenetic clocks may be better proxies for the presumed accelerated aging rate in SZ. Here we investigate 14 epigenetic clocks using three publicly available DNAm datasets from whole blood, comparing SZ to non-psychiatric controls (NPC). In all data sets, we find SZ age acceleration in three clocks previously shown to be most predictive of age-related morbidity and mortality risk. In contrast, two clocks developed to capture mitotic rate are decelerated in SZ, consistent with low cancer rates despite smoking observed in epidemiological studies of SZ. We use these clocks to investigate the determinants of altered aging in SZ, such as smoking, alcohol, BMI, age-associated proteins, blood cell composition, and psychotropic medications. Principal component analysis suggests mortality clock acceleration, mitotic clock deceleration, and medication effects are independent phenomena in SZ. Our study demonstrates the importance of studying the various epigenetic clocks in tandem and highlights their potential utility for understanding how mental illness influences long-term outcomes including cancer and early mortality.

